# Mate choice decision rules: Trait synergisms and preference shifts

**DOI:** 10.1002/ece3.3831

**Published:** 2018-01-29

**Authors:** Nancy Tyler Burley, Elnaz Hamedani, Cole Symanski

**Affiliations:** ^1^ Department of Ecology and Evolutionary Biology University of California, Irvine Irvine CA USA; ^2^ Department of Entomology University of California Riverside CA USA

**Keywords:** linchpin trait, mate choice, preference shift, secondary sexual traits, trait synergism, zebra finch

## Abstract

An important and understudied question in sexual selection is how females evaluate information from multiple secondary sexual traits (SSTs), particularly when expression of traits is phenotypically uncorrelated. We performed mate choice experiments on zebra finches (*Taeniopygia guttata castanotis* Gould) to evaluate two hypotheses: preference shifts (obstacles to choice using one trait increase chooser reliance on others) and trait synergisms (choice based on the sum/product of two or more independently varying traits). The first experiment, which employed males raised on diets that impact SST expression, supported the trait synergism hypothesis: overall, male pairing success was best predicted by synergisms involving beak color and cheek patch size. Results did not support the preference shift hypothesis. Results of a follow‐up experiment that included males reared on a single diet, and in which male beak color and cheek patch size were manipulated, were also consistent with the trait synergism hypothesis. Results have implications for understanding the long‐term persistence of multiple SSTs in populations and for the measurement of repeatability and heritability of mate preferences.

## 1 INTRODUCTION

Over the last several decades, researchers have focused much attention on ornamental signals, or traits that evolve and are maintained through the mate choice component of sexual selection. Major questions of interest have included the relative roles of intrasexual and intersexual selection in secondary sexual trait (SST) evolution (Andersson, Pryke, Ornborg, Lawes, & Andersson, 2002; Santos, Scheck, & Nakagawa, 2011); the information value of such signals (Andersson & Simmons, 2006; Zahavi, 1975), including the extent to which multiple ornaments provide independent versus redundant information on male quality (Hill, 2011; Johnstone, 1996; Laucht & Dale, 2012; Møller & Pomiankowski, 1993); and possible costs, constraints and trade‐offs in ornament development and expression (Hebets & Papaj, 2005; Wagner, Beckers, Tolle, & Basolo, 2012), including the hypothesis that the cost to females of engaging in complex mate choice limits ornament number (Candolin, 2003; Iwasa & Pomiankowski, 1994).

In contrast to the above questions, the puzzle of how females may weigh the potentially conflicting information that multiple male ornaments provide in order to reach a mating decision has received limited attention (Candolin, 2003). One possibility is that females change their focus from one trait to another as circumstances dictate. For example, females might focus exclusive attention on whichever trait has the greatest local variability (Calkins & Burley, 2003; Reid & Weatherhead, 1990) or signals information most relevant to current ecological conditions (Chaine & Lyon, 2008). Within populations, individual choosers may select mates using different traits (Brooks & Endler, 2001; Murphy & Gerhardt, 2000), either as the result of variation in sensory processing ability (Henry, Gall, Bidelman, & Lucas, 2011; Ronald, Fernandez‐Juricic, & Lucas, 2012), or from variation in costs and benefits associated with mating with particular phenotypes (Atwell & Wagner, 2015; Burley & Foster, 2006; Widemo & Sæther, 1999). Modeling suggests that multiple ornaments could be maintained in populations when an individual choice is based on a single trait yet choosers base selection on different traits (Wagner et al., 2012). Nevertheless, females may frequently select mates using information from more than one ornament, as well as using nonornamental cues (Andersson, 1994; Candolin, 2003; Jennions & Petrie, 1997).

In broad terms, multiple ornaments may be evaluated either sequentially or simultaneously (reviewed in Castellano, Cadeddu, & Cermelli, 2012). Sequential choice occurs, for example, where males advertise for mates using both long‐distance signals such as acoustic traits and short‐distance signals, such as visual displays (Gibson, 1996). In this case, only males that meet a female's standards for one trait are assessed for additional traits. Where multiple, independently varying traits are assessed concurrently, various mate assessment rules may be employed (Candolin, 2003; Rowe & Skelhorn, 2004). Two such rules are explored here: A *preference shift* occurs when obstacles to choice based on a particular trait cause females to increase their relative emphasis on other traits; potential obstacles include low trait variability and ecological conditions that decrease chooser ability to perceive variation (Green, Osmond, Double, & Cockburn, 2000; Zuk, Ligon, & Thornhill, 1992). *Trait synergism* occurs when females base preference on some combination of two or more traits. Typically such preferences are assumed to be additive across traits (Cole & Endler, 2015), but several studies suggest that choice may be based on the product of values of two or more traits (Höglund, Alatalo, Lundberg, & Ratti, 1994; Møller, Saino, Taramino, Galeotti, & Ferrario, 1998; Rybak, Sureau, & Aubin, 2002). To the extent that interactions between two or more traits influence male attractiveness, experimental demonstration of preference for any one trait may depend on inclusion of all relevant traits in a single study (Galván, 2010; Hamilton & Sullivan, 2005).

**Figure 1:**
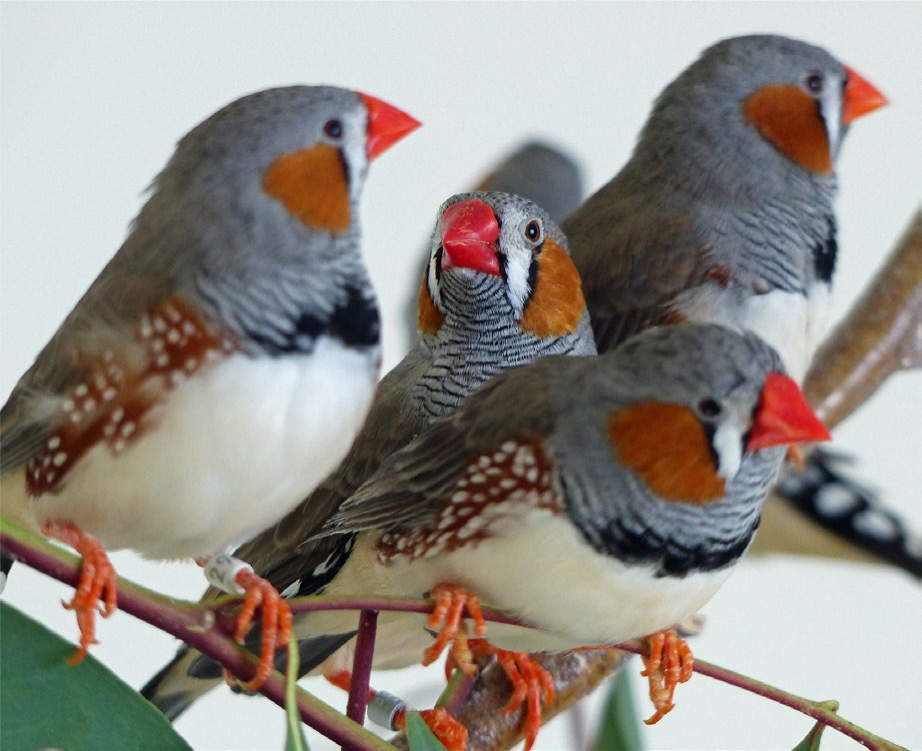
Adult male zebra finches, illustrating variable beak color and cheek patch size. The central bird has the reddest beak and the largest cheek patches; the bird on the back right has the least red beak, while the bird on the left has the smallest cheek patch size of the four shown here. Photograph by Nancy Burley

One way to explore how organisms evaluate complex sets of information is to compare the success of statistical models based on different assumptions about how choices are made in predicting results of mate choice experiments. Here we use this approach to investigate mate choice by female zebra finches (*Taeniopygia guttata castanotis* Gould; Figure 1) for several SSTs for which one or more previous studies have reported evidence of female mate preferences: beak color (beaks that are redder and darker are preferred over more orange/lighter beaks: Burley & Coopersmith, 1987; Simons & Verhulst, 2011); the size of the chestnut cheek patch (larger cheek patches preferred: Tschirren, Postma, Rutstein, & Griffith, 2012); and two song traits, namely the number of syllables in a male's song motif (a greater number preferred) and motif duration (longer duration preferred) (Holveck & Riebel, 2007; Spencer, Buchanan, Goldsmith, & Catchpole, 2003; Spencer et al., 2005). Where multiple studies have investigated female choice for these traits, differing results have often been obtained (see Simons and Verhulst (2011) for beak color, Riebel, 2009 for song traits). Yet to our knowledge, no experiment has reported simultaneous choice for more than one visual trait, or any combination of visual and acoustic traits, by a single cohort of females of this species.

A likely contributor to the contrasting results of various studies is between‐population variation in trait distributions due to genetic effects. Also, in laboratory populations held under favorable conditions, there may be low variability in expression of condition‐dependent traits, which would limit opportunity for expression of mate preferences for them. In the first of two experiments reported here, we reared and maintained males on two different diets to increase SST variation in our captive population: males were either provisioned with a basic diet of grass seed, or with daily supplements of hen's egg in addition to their seed diet. This manipulation was expected to influence the song traits under study (Spencer et al., 2003, 2005) due to variation in nutritional stress during development (Nowicki, Peters, & Podos, 1998); motifs of longer duration/greater number of syllables were expected in the supplemented diet. The diet manipulation was also expected to impact size of the chestnut‐colored cheek patch, a pheomelanin‐based plumage trait (McGraw & Wakamatsu, 2004), because the supplemented diet treatment had greater availability of methionine (Allen & Hume, 1997), a precursor in the metabolic pathway for pheomelanin production (Galván & Solano, 2009). Rearing diet might also impact adult beak color, as cholesterol supplementation has been reported to increase redness of male beaks (McGraw & Parker, 2006); however, healthy adult males may show relatively little variation in beak color expression when breeding opportunities are unavailable for a substantial interval (Burley, Price, & Zann, 1992). Due to anticipated differences in SSTs of males reared on experimental diets, we thus expected that females would tend to prefer males reared on the supplemented diet.

In our first experiment, female choosers were exposed to a small choice set of males and given 5 days to choose mates. Statistical analysis illuminated the extent to which males’ relative (ranked) trait values within the choice sets in which they participated predicted pairing success. Because the validity of this methodology relies on the untested assumption that females regularly choose among a limited set of options available (a relative choice criterion), rather than using an absolute or threshold criterion of acceptability (Andersson, 1994; Castellano et al., 2012; Dale & Slagsvold, 1996; Rybak et al., 2002), we compared results based on relative trait values with analyses based on absolute trait values. We tested the preference shift hypothesis by asking whether females de‐emphasized choice for male beak color (the least variable trait in this sample, and the SST best documented to influence male mate‐getting) and instead emphasized other SSTs when tested with those sets with lower beak color variability. To investigate the possibility of choice based on trait synergism, we included models in which scores for traits found to contribute to male pairing success were combined additively and multiplicatively. Finally, we examined the influence of rearing diet on SST expression and male pairing success.

A second experiment was performed to assess female preference for beak color and cheek patch variation among males raised on an intermediate diet. This experiment involved manipulation of both traits under study and was undertaken to further clarify whether preferences observed in the first experiment were caused by diet treatment differences among males or were the result of preferences for SSTs per se. In addition, this experiment provided an additional test of the trait synergism hypothesis.

## 2 METHODS

All experiments were carried out in accordance with the Institutional Animal Care and Use Committee at the University of California, Irvine (IACUC protocol 1998‐1334) and were consistent with USA federal guidelines.

### 2.1 Study species and rearing of participants

The zebra finch is a socially monogamous species that shows bidirectional mate choice (Burley & Coopersmith, 1987; Jones, Monaghan, & Nager, 2001) and typically pairs for life. Nesting takes place in colonies of variable size, and birds defend only the immediate surroundings of their nest (Zann, 1996).

Subjects were derived from an outcrossed laboratory colony (effective population size ≥120 birds) of domesticated zebra finches. All participants and their parents had wild‐type plumage and conformation. Only birds judged to be in good condition were employed in experiments.

Males employed in Experiment 1 were cage‐reared indoors by parents that had been allowed to choose mates in flocks containing 60 adults. The parental generation had been reared and maintained on a standard (LAB) diet, which includes ad libitum access to a commercial grass seed mix, as well as calcium and mineral supplements; green vegetable and cooked hen's egg are also included in this diet, each offered three times per week on alternating days. When selected for the current experiment, pairs were moved indoors and maintained on a 14L:10D photoperiod under full‐spectrum illumination. Following a 2‐week acclimation, pairs were randomly assigned a breeding diet treatment; a nest was added to their cage 2 weeks later, and the first female began laying 5 days later. Breeding diets were identical to the LAB diet, with the following exceptions: the basic seed (lower‐quality, or LO) diet provided no egg, while the supplemented (higher‐quality, or HI) diet provided daily egg access. Initially, each pair on the HI diet received four grams of egg daily, with the amount of egg increasing over the nesting cycle in proportion to total brood mass (maximum egg allocation = 2 g bird^−1^ day^−1^).

When the youngest sibling reached 35 days of age, offspring were removed from their parents’ cage and housed with brood members on their natal diet. (Females were removed from sibling groups when plumage indicators of sex became apparent, at about 42 days of age.) For the next 30 days, young birds were exposed at close quarters to six different pairs of adult zebra finches held in adjoining cages, with adult pairs used in rotation to tutor males on both diets. This procedure was implemented to standardize exposure of subjects to conspecific adult phenotypes during the period of sexual imprinting and song learning (Böhner, 1990; ten Cate, Los, & Schilperoord, 1984). At 70–80 days of age, males from multiple sibships on the same diet were consolidated into larger cages at a standard holding density.

One month before the start of the experiment, phenotype measurements (see below) were made of 40 males assigned to the first experiment; then they were released into a flight to allow their flight muscles to become conditioned. At this time, all birds were placed on the LO diet and maintained on it for the remainder of the experiment. This procedure was implemented as a guard against the possibility that females choose mates based on their current/recent diet (perhaps by olfactory means—e.g., Golűke, Doerrenberg, Krause, & Caspers, 2016). Cheek size measurements made before the diet transition were later found not to differ from those taken when males were first employed in trials (*t* test, Cohen's *d* = −.015); beak color scores increased during this interval (*t* test: Cohen's *d* = −.608), most likely due to the reduced housing density of birds (Burley et al., 1992).

Females used as choosers of the first experiment, and both sexes of birds involved in the second experiment were colony‐reared on the LAB diet. In both experiments, males and females in any given choice trial were not closely related (coefficient of relatedness <0.125); birds had no prior social experience with the opposite‐sex individuals use in the same trial. During trials, participants were color‐banded for individual identification with colors previously demonstrated to not influence mating attractiveness (Burley, 1985).

### 2.2 Pairing trials (Experiment 1)

Trials were conducted in flights (3 m × 3 m × 2.8 m) outfitted to minimize intrasexual interference competition. Each wall of the test arena contained a single metal grid or “nest tier,” subdivided into nine cubicles suitable for nesting. Translucent plastic sheets suspended from the ceiling provided partial visual isolation of birds attending adjacent tiers. Seed and water were available ad libitum, and nesting material and perches were abundant.

Male test sets were composed of four individuals, two from each diet treatment. Sets were created without reference to SSTs under study. Within sets, birds were matched for body size (within 1.5 g) to reduce any size‐related advantage during intrasexual interactions. All males included in analysis of pairing success participated in two choice sets; each male was tested in a unique combination of sets, and no two males participated in more than one set together. Trials involving a given male were spaced three or more weeks apart. (Preliminary tests had established males regularly sought new mates after this interval.) Male pairing success was defined as the number of trials (0, 1, or 2) in which mates were acquired.

Two adult female choosers were presented with each male test set. Each female participated in a single trial regardless of whether or not she made a choice.

The trial procedure involved releasing a set of males into the test arena at 1200 hr on day 0; females were released 24 hr later; birds were removed from the arena late on the morning of day 6. During preliminary trials, males showed only affiliative activities on day 0, and both sexes had limited interactions on day 1. We therefore focused sampling effort starting day 2. On days 2 through 5 (and, if needed, on day 6; see below), two sampling procedures were carried out between 0800 and 1300 hr: a 10‐min all‐accounts sampling of male behavior and 10‐min focal samples of each female's social behavior. In the all‐accounts sample, males were scored for agonistic interactions and attendance at nest sites. During focal samples, a record was made of all interactions between the sexes, including those scored as instantaneous events (courtship/copulation, traveling together) and those that were timed (huddling, allopreening, attendance at a nest site). At the end of each focal sample, the observer recorded the percentage of the sample the female had affiliated with each male.

Female choice of a particular male was scored when all of the following pairing criteria were met during at least two observation sessions: (1) she spent ≥50% of the sample affiliating with the same male; (2) she engaged in one or more of the following contact activities with that male: courtship/copulation, allopreening and/or huddling; and (3) she spent little time affiliating with other birds (≤10% of the sample) and did not engage in affiliative contact activity with any bird other than the chosen male. The day that a female was first observed to reach this set of criteria is dubbed “first affiliation.” When first affiliation occurred on day 5, the female was sampled the next morning to provide an opportunity to reach the choice criterion. If a female reached the choice criterion with a given male early in the week but did not continue to associate with that male throughout the trial, the male was not scored as having paired.

Trials were aborted if any of the six birds showed a tendency to initiate or accept affiliation by a member of the same sex. (Same‐sex affiliation can result from sustained housing under single‐sex conditions—Butterfield, 1970; Zann, 1996.) Birds scored as participating in same‐sex affiliation were not retested. At the completion of each trial, all birds were inspected for feather loss and signs of injury.

Intrasexual interference competition is not a prominent aspect of the mating system of this species (Zann, 1996), but agonistic interactions among males might interfere with female choice during trials (e.g., if despotic relationships precluded/discouraged females from pairing with less aggressive males); alternatively, such interactions may reflect the choices being made (e.g., if males became objects of aggression when they attempted to affiliate with females that were associating with other males). As mating patterns based on both intrasexual and intersexual selection can predict a positive relationship between male tendency to initiate aggression and succeed in mate‐getting, we developed alternative predictions to aid interpretation of the significance of intrasexual aggression in these trials: If interference competition exerted a strong influence on trial outcome, we would observe periods of intense competition among males, including successful efforts to limit others’ access to nesting sites, and a negative correlation between male tendencies to initiate and be the object of intrasexual aggression. However, if female choice were the predominant influence on trial outcome, individual males would not prevent others from obtaining nest sites access, nor would there be a negative relationship between male tendency to initiate and be the object of aggression.

### 2.3 Preference trials (Experiment 2)

In this experiment, beak color and cheek patch size were manipulated in order to separate preference for these traits (observed in the first experiment) from preference for possible trait correlates, especially rearing diet. To accomplish this, young adult males whose beaks had not yet reached maximum expression were employed; this enabled experimenters to realistically enhance beak color by application of translucent colored markers (Burley & Coopersmith, 1987). Prior to enhancement, the beaks of these males were orange or red‐orange and contained no trace of the gray or black coloration typical of immature birds. Trials were conducted in the same arenas as the first experiment and were of similar design. However, pair formation was not established; rather, trials proceeded only to the stage of “first affiliation.” The rationale for this change of procedure and additional differences between the experiments are summarized below.

Male test sets were composed of three males matched for several traits (mass [within 1.5 g], beak color, and cheek patch size) as closely as possible given the size of the pool of available birds. Two males per set were randomly assigned to have their beak color expression made more red by the application of nontoxic, unscented red marker (Crayola, Inc: Easton, PA, USA); as a control, the third male's beak was treated with orange marker, which enhanced color expression to a smaller degree. One of the two males assigned to have reddened beaks was also assigned to have his cheek patch size reduced approximately 15% by trimming the ends of the feathers at the margins of the cheek patch; as a control, the very tips (≃½ mm) of these feathers were also trimmed on the remaining two birds. After trait manipulations, each test set consisted of a red‐orange‐beaked male with a larger cheek patch (O+), a red‐beaked male with a smaller cheek patch (R−), and a red‐beaked male with a larger cheek patch (R+). A logical fourth treatment group (O−) was not included for three reasons. (1) It would have required a much larger subject pool to create well‐matched sets consisting of four males. (2) In mate choice trials, the degree of male–male interference competition increases with the sex ratio (percent male), which obstructs the goal of scoring female choice. (3) As previous work on this species indicates that mate preferences are transitive (sensu N. T. Burley, unpublished data; Burley & Coopersmith, 1987; Navarick & Fantino, 1972), the benefit of including a treatment group expected to be the least preferred is more than offset by the costs of doing so. All postmanipulation phenotypes were within the naturally occurring adult range. Each male participated in a single test set.

Two female choosers were presented with each set. Females that expressed a preference in their first trial were not retested. Most of those that did not express a preference were given one additional opportunity to do so in a subsequent trial. The rationale for retesting females was that these trials were of short duration, and mate selectivity (Burley & Foster, 2006; Forstmeier, Coltman, & Birkhead, 2004; Jennions & Petrie, 1997) may have differed between females that were slow‐to‐choose versus those that were fast‐to‐choose.

The trial procedure differed from that described for Experiment 1 in several significant ways. Trial duration was variable, ending after the first female in a set reached the “first affiliation” criteria. If two females reached the criteria during the same observation session, both of their preferences were scored; otherwise, the trial resulted in a single preference being displayed. This procedure was adopted because the experiment's design allowed only one female full choice among the three male phenotype options. (Preliminary trials performed during the development of the design included a single chooser female with the set of three males. Unfortunately, in this setting, male–male competition escalated to a level that was much higher than observed in the previous experiment.)

To increase understanding of the role of male aggression in trial outcome, observations began on the day the males were released into the arena (Day 0) and were performed twice daily until one female reached the affiliation criterion. Two observation sessions took place each day (the first between 7:30 and 11:30 a.m.; the second, between 12 and 4 p.m.). A trial was suspended after 1 week if neither female reached the affiliation criterion.

During trials, males were caught by hand net three times daily to have their beak color touched up: before the a.m. observation session; between the a.m. and p.m. sessions, and after the p.m. session.

### 2.4 Phenotype measurements

Male beak color was scored using the Munsell^®^ Book of Color, glossy finish collection (X‐rite Pantone: Carlstadt, NJ, USA). This system assigns separate numerical scores to hue, value, and chroma of color. Scoring was performed under standard illumination 1‐to‐3 days before trial start. Prior to analysis, Munsell^®^ scores were transformed into a single index that results in a higher score for colors that are redder, darker, and brighter (more saturated). Previous research found that female zebra finches prefer males that score higher on this index (Burley & Coopersmith, 1987), and spectrophotometer‐based scores have been found to correlate well with Munsell^®^ scores (Bolund, Schielzeth, & Forstmeier, 2010). (Male beak color shows limited UV reflectance—Bolund et al., 2010.)

Body size measurements (mass, and in Experiment 1, tarsus length) were made when beak color was measured.

Cheek patch size was measured differently in the two experiments. In Experiment 1, one person held the bird with the cheek horizontal and the cheek feathers in relaxed posture, while a second person scored size by holding a transparent grid over the cheek patch and counting the number of squares overlying chestnut‐colored feathers; the value obtained was subsequently transformed to area (mm^2^). Cheek patch size was measured twice, 6 weeks apart, and is reported as the average of four measurements (2 cheek patches per bird, each measured twice). In Experiment 2, measurements were made in ImageJ from photographs taken before and after cheek patches were trimmed; during photography, birds were hand‐held as described for Experiment 1. Authors have found good correspondence between the measurement protocols (unpublished data). Repeatability estimates of beak color and cheek patch measurements vary between 0.88 and 0.95 among samples.

Courtship (“directed”) songs were recorded at the end of Experiment 1 in a sound attenuation chamber, using a high‐fidelity microphone (Audio Technica model AT 2020) and Garage Band software. Songs were imaged and scored in Song Analysis Pro© (Tchernichovski & Mitra, 2004). Male zebra finches produce a single, highly stereotyped song motif (Zann, 1996). Here, song traits were based on average values from a sample of five motifs, with introductory elements excluded (Zann, 1996). Syllables were identified as units of sound surrounded by silence (Holveck & Riebel, 2007); motif duration was measured from the start of the first syllable to the end of the last syllable. Songs of three of the 40 males originally assigned to this experiment were not recorded (one had died and two failed to sing in the recording chamber); none of these males participated in two trials.

All measurements involving birds in Experiment 1 were made without reference to males’ rearing diet.

### 2.5 Statistical analyses

In Experiment 1, *t* tests were performed to identify diet effects on aspects of phenotype for the 40 males originally assigned to the experiment's pool; effect size was estimated by Cohen's *d* (Cohen, 1988). Correlations in trait expression were assessed by Pearson's (*r*) test. A principal components analysis (PCA) was performed on two intercorrelated size traits (mass and tarsus length). Both traits loaded positively on the first principal component, which accounted for 65% of the variance; this measure was used as a body size index. A separate PCA analysis was performed for the 28 males that participated in two successful trials; here the first principal component accounted for 74% of the variance.

Reverse, stepwise procedures were used to evaluate which SSTs contributed to mate choice decisions in Experiment 1. The use of stepwise procedures has been critiqued on the grounds that it facilitates the undisciplined search for pattern (Burnham & Anderson, 2002). Here this problem is offset by the following considerations: (1) we developed specific a priori hypotheses and predictions for testing; (2) we delineated a small set of models for analysis by employing a limited number of SSTs as predictor variables, all of which have been found to predict male zebra finch mate attractiveness in at least one published study; (3) we combined the use of stepwise procedures with other quantitative approaches, as well as conducted multiple experiments, to assess pattern coherence. Collectively, our analyses reduce the chances of type 1 error and reliance on a single best model, which are additional problems attributed to stepwise procedures (Hegyi & Garamszegi, 2011; Whittingham, Stephens, Bradbury, & Freckleton, 2006).

Analyses to identify predictors of pairing success (number of times a male was scored as having paired) included all males that participated in two successful trials. Influences of rearing diet and size were explored in an ordered logistic regression model (Long & Freese, 2006), with pairing success as the dependent variable. Any variable not contributing to the model (*p* > .15) was removed in a reverse, stepwise procedure. After the final model was obtained, individual excluded variables were re‐entered to assess whether they had been inappropriately removed (Hegyi & Laczi, 2015).

Models to measure contributions of ranks of SSTs to pairing success employed Kendall's correlation test (tau‐*a*; Newson, 2002). In these tests, a male's trait rank is the average of his ranks from the two test sets in which he participated. Initially, separate tests were performed for each trait under study (beak score, cheek score, syllable number and song duration). (Although the two song traits were positively correlated, they were considered individually—rather than collapsed into principal components—to maximize comparability with previous studies.) To explore the possibility of trait synergisms, Kendall's test was then repeated using trait rank sums and products; these tests included only traits that had shown a tendency (*p* ≤ .15) to correlate with pairing success. Relative model performance was assessed by the magnitude of the normal (*z*) approximation.

To test the preference shift hypothesis, males were partitioned into two groups based on whether the average range of beak score in the two test sets in which they participated was below or equal to/above the median range of all test sets. The correlation procedure outlined in the previous paragraph was then repeated for each group.

To assess contribution of absolute SST scores to pairing success, ordered logistic regression models (Long & Freese, 2006) were performed. Prior to analysis, a two‐step transformation was applied to trait values: first, scores for each variable were *z*‐transformed ([individual trait score − mean trait score]/standard deviation of trait score) to achieve the same means (0) and similar variances; next, all scores for each trait were made positive by the addition of a constant that resulted in the minimum score of one. The first multiple‐trait model included all four SSTs, with a reverse stepwise procedure (*p*‐to‐remove = .15) used to achieve a final (“best”) model. Then, for traits that entered the best additive model (model 15), the model was re‐run with the inclusion of the product of those trait values (model 16), and the stepwise procedure repeated. Relative model performance was assessed as the best final model produced by the reverse stepwise procedure (model 17). In these models, the parallel regression assumption was validated using Brant's test (Long & Freese, 2006). Logistic regression models included odds ratios, with corresponding confidence limits.

As the purpose of presenting a series of models exploring contributions of SSTs to male pairing success was to compare their outcomes, each model is reported without correction for multiple comparisons. In addition, each table summarizing results of this modeling procedure identifies which of the models remain statistically significant (*p* ≤ .05) after sequential Bonferroni correction.

Nonparametric tests were used to investigate the influence of male–male aggression on the outcome of pairing trials because residuals of parametric tests could not be normalized in some cases. Spearman correlation tests were used to investigate the relationship between a male's pairing success score (fraction of trials in which a male paired) and his average per‐sample incidence of initiating aggression, as well as between the average incidence of initiating and being an object of aggression. Spearman tests were also used to determine whether SST expression correlated with aggression scores. In separate analyses, we asked whether aggression scores differed between males reared on the two diets (Kruskal–Wallis [KW] *H* tests). Goodman and Kruskal's test (Ghent, 1976) was used to quantify the association between within‐trial ranks of male SSTs and absolute trait scores.

In Experiment 2, linear mixed models (LMMs) were performed to examine phenotype differences of males included in test sets. Specifically, we asked whether beak score and cheek patch size (dependent variables) varied across treatments (O+ = treatment 1; R+ = treatment 3) just prior to and after phenotype manipulations; male set number was included as a random effect. KW *H* tests were performed on male aggression variables. To maximize comparability with Experiment 1, separate analyses were performed for day 0 samples (only males present in arena), day 1 (first 24 hr after the addition of females) and across all remaining samples. Spearman correlation tests were used to assess predictions concerning male aggression. Female preference was assessed by applying a chi‐square test with Yates’ correction to the number of males of each phenotype preferred/not preferred across all trials.

For both experiments, mean values are reported with standard errors (*SE*); median values are reported for results analyzed by nonparametric tests. Where corrected by sequential Bonferroni tests for multiple comparisons, probability values are reported as “corrected”; otherwise, the values have not been corrected. Analyses were performed in STATA 14.

## 3 RESULTS—EXPERIMENT 1

Of the 40 males originally assigned to the experiment's pool, 28 completed two trials. The remaining 12 males were assigned to only one trial each. Six males were dropped after observation of same‐sex affiliations; in addition, one male died before his second trial, another showed a substantial loss of condition, and four males were not assigned to a second trial due to lack of availability of size‐matched set members at the end of the experiment. Male age at the time of first test averaged 388 (±24) days and showed no treatment difference (Cohen's *d *=* *.215, *p* = .57). Of the 28 males included in analysis, none was observed to reject affiliation by females.

### 3.1 Diet effects on phenotype

For the 40 males in the experiment's pool, two SSTs showed diet effects: cheek patch size was larger, and motif duration longer, among HI‐diet males (Table 1). Body size PC1 did not differ based on diet history (Table 1). Bird age did not influence SSTs or body size (all *p*s > 0.30).

**Table 1:** Trait expression of males reared on lower‐quality (LO) versus higher‐quality (HI) diets (Experiment 1)

Trait	Experiment pool		*p* a	*d* a	95% CI	Males completing 2 pairing trials (*N*s = 14)	
	LO	HI				LO	HI
	*X* ± *SE* (*N*)	*X* ± *SE* (*N*)				*X* ± *SE*	*X* ± *SE*
Size PC1	−0.03 ± 0.28 (20)	0.03 ± 0.24 (20)	.86	−.06	−0.67 to 0.56	0.10 ± 0.33	−0.10 ± 0.33
Beak color	26.1 ± 0.1 (20)	26.1 ± 0.1 (20)	.66	−.14	−0.76 to 0.48	26.0 ± 0.1	26.1 ± 0.1
Cheek size^b^ (mm^2^)	99 ± 2 (20)	112 ± 2 (20)	.001	−1.4	−2.11 to −0.71	100 ± 2	112 ± 3
Syllable number	4.8 ± 0.4 (19)	5.7 ± 0.5 (18)	.17	−.46	−1.11 to 0.20	4.7 ± 0.5	6.1 ± 0.5
Motif duration^b^ (ms)	437 ± 30 (19)	582 ± 45 (18)	.01	−.89	−1.56 to −0.21	416 ± 33	615 ± 55

a *t* Test two‐tailed *p*s (uncorrected); *d* = Cohen's effect size estimate with 95% CI.

b Indicates comparison remains statistically significant after sequential Bonferroni test.

For males that participated in two successful trials, trait expression was representative of the larger pool from which they were drawn (Table 1). The two size traits (mass and tarsus length) were significantly correlated (Pearson *r* = .48, corrected *p* = .009), as were the two song traits (syllable number and song length: *r* = .66, corrected *p* = .0006). No other traits were significantly intercorrelated (uncorrected *p*s > .20).

### 3.2 Effects of diet and SSTs on pairing success

**Figure 2:**
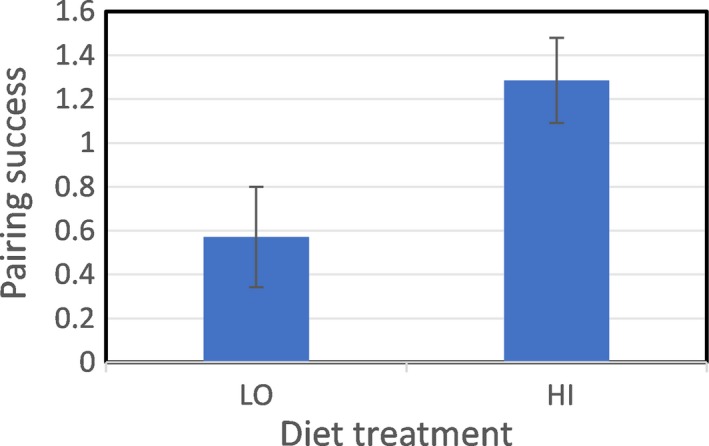
Pairing success of males (mean number of times chosen across two trials ±*SE*) in Experiment 1 as a function of rearing diet (ordered logistic regression: LR χ^2^ [1 *df*] = 5.57, odds ratio = 5.71 (confidence limits [1.26–25.89], *N* = 28, *p* = .018)

Pairing success differed by diet treatment: HI‐diet males had higher pairing success (Figure 2). For all pairing events (*N* = 28) involving males that completed two trials, the interval to reach the pairing criterion averaged 5.0 (± 0.2) days and did not differ between diets (*p* = .71). Size PC1 did not contribute to pairing success (ordered logistic regression, *p* = .26).

Of the four single‐trait correlation tests based on trait ranks, only one trait reached statistical significance in the full data set: rank of cheek score was positively correlated with pairing success (Table 2). Two traits—cheek score and beak score—reached the criterion for inclusion in models based on combined trait models. Both the additive and product score models (models 5 and 6 in Table 2) were highly significant and remained so after correction for multiple comparisons: males that had both large cheeks and high beak scores were more likely to display success in pairing trials.

**Table 2:** Models predicting male pairing success (Experiment 1) based on relative (within‐set) secondary sexual trait expression: full sample (*N* = 28)

Model no.	Variable	τ_*a*_ ± *SE*	*z*	*p*	95% CI
1	Cheek patch size	.243 ± 0.123	1.98	.048	0.002–0.484
2	Beak color	.201 ± 0.133	1.52	.129	−0.059 to 0.461
3	Syllable number	.116 ± 0.101	1.15	.250	−0.082 to 0.315
4	Motif duration	.092 ± 0.128	0.73	.467	−0.157 to 0.342
5	(Cheek + beak) score^a^	.312 ± 0.119	2.62	.009	0.079–0.546
6	(Cheek × beak) score^a^	.352 ± 0.118	2.99	.003	0.121–0.583

Kendall's tau‐*a*,* z* approximation with 95% CI.

a Indicates model remains statistically significant after sequential Bonferroni test.

**Figure 3:**
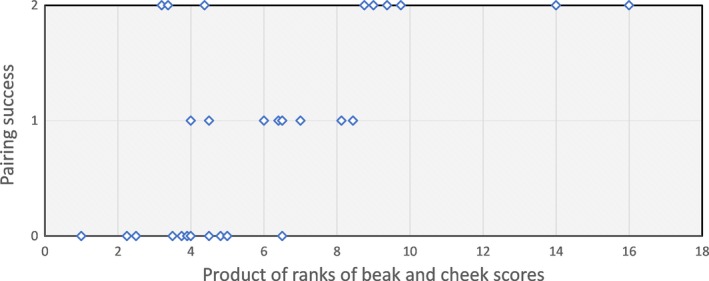
Pairing success of males participating in trials with greater variation in beak color in Experiment 1: results of model 12 in Table 3 (product of beak color and cheek size ranks; Kendall's tau‐*a*:* z* = 2.99, *p* = .003)

For the males from sets with low variation in beak color, no individual trait showed a tendency to influence pairing success (*p*s > .5), so no combined trait analysis was performed. For the males from sets with higher beak score variation, ranks of three traits—beak score, cheek score, and syllable number—met the criterion for inclusion in combined trait models. All four combined trait models were significant and stronger than any model based on a single trait (Table 3); the strongest model was that based on product of cheek and beak score ranks (model 12; Figure 3).

**Table 3:** Models predicting male pairing success (Experiment 1) based on relative (within‐set) secondary sexual trait expression: males from sets with high beak score variation (*N*= 15)

Model no.	Variable	τ_*a*_ ± *SE*	*z*	*p*	95% CI
7	Cheek patch size	.343 ± 0.183	1.87	.062	−0.017 to 0.702
8	Beak color^a^	.467 ± 0.168	2.78	.005	0.138–0.796
9	Syllable number^a^	.324 ± 0.133	2.43	.015	0.138–0.795
10	Motif duration	.248 ± 0.176	1.41	.159	−0.097 to 0.592
11	(Cheek + beak) score^a^	.543 ± 0.105	5.18	.000	0.337–0.748
12	(Cheek × beak) score^a^	.638 ± 0.068	9.39	.000	0.505–0.771
13	(Cheek + beak + syllable) score^a^	.448 ± 0.122	3.66	.000	0.208–0.688
14	(Cheek × beak × syllable) score^a^	.512 ± 0.139	3.69	.000	0.241–0.787

Kendall's tau‐*a*,* z* approximation with 95% CI.

a Indicates model remains statistically significant after sequential Bonferroni test.

**Figure 4:**
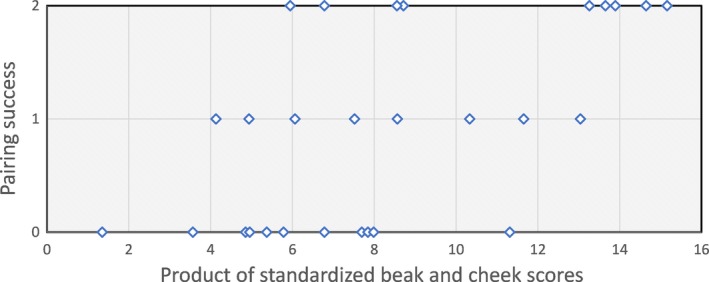
Pairing success of all males based on absolute trait values in Experiment 1: results of the final model (model 17 in Table 4; LR χ^2^ (1) = 10.80, *N* = 28, model *p* = .001, pseudo *R* ^2^ = .177)

Models that employed absolute trait scores achieved similar results (complete male sample only; Table 4). The best additive model included cheek score and beak score (model 15); contributions of song traits did not approach significance (*p*s > .4). In the model that included the cheek score, beak score, and the product of these trait values, the beak × cheek product score was the only variable that approached significance (model 16); a likelihood ratio (LR) test indicated that model 16 is better than model 15 (LR χ^2^ (1) = 3.93, *p* = .047). When the stepwise procedure was applied to model 16, only the product score remained (model 17) (LR χ^2^ test for difference between model 16 and 17 = 1.26, *p* = .53). The final model (model 17; Figure 4) produces the tightest range of confidence limits of odds ratios and is the only model to exclude variables that have a lower confidence limit of ≤ 1.

**Table 4:** Best models of secondary sexual trait influence on pairing success (Experiment 1) using absolute trait scores. Models include odds ratios with corresponding confidence limits

Model no.	Type	Trait	Coefficient ± *SE*	*z*	*p*	Odds ratio	95% CI
15	Additive model	Cheek score	0.988 ± 0.423	2.34	.019	2.686 ± 1.136	0.930–4.948
		Beak score	0.763 ± 0.427	1.79	.074	2.145 ± 0.915	1.173–6.151
16	Multiplicative model	Cheek score	−1.276 ± 1.232	−1.04	.300	0.279 ± 0.344	0.025–3.124
		Beak score	−1.154 ± 1.036	−1.11	.265	0.315 ± 0.327	0.041–2.404
		Cheek × beak	2.885 ± 1.521	1.90	.058	17.897 ± 27.226	0.908–352.945
17	Simplified						
	Multiplicative model	Cheek × beak	1.340 ± 0.456	2.94	.003	3.820 ± 1.360	1.563–9.334
Model 15^a^: ordered logistic regression: LR χ^2^ (2) = 8.12, *N* = 28, model *p* = .017, pseudo *R* ^2^ = .133							
Model 16^a^: ordered logistic regression: LR χ^2^ (1) = 12.06, *N* = 28, model *p* = .007, pseudo *R* ^2^ = .198							
Model 17^a^: ordered logistic regression: LR χ^2^ (1) = 10.80, *N* = 28, model *p* = .001, pseudo *R* ^2^ = .177							

a Indicates model remains statistically significant after sequential Bonferroni test.

For all four SSTs, the average ranked scores were significantly associated with corresponding absolute scores (Goodman and Kruskal's tests, *t* ≥ 3.97, *p*s ≤ .001); the sum and product of cheek and beak score ranks and the corresponding z‐transformed sum and product scores were also correlated (*t* ≥ 3.19, *p*s ≤ .01).

### 3.3 Intrasexual aggression

The median number of aggressive interactions initiated by males was 0.70 (±0.07) per sample. The two indices of male aggression (initiator vs. object) were positively correlated (Spearman ρ = .45, *p* = .03). Male–male aggression frequency was positively correlated with pairing success (ρ = .55, *p* = .005). No SST score, whether based on within‐trial rank or on absolute value (and including cheek/beak sum and product scores), was significantly correlated with either male tendency to be initiator or object of aggression (a total of 24 tests; all uncorrected *p*s ≥ .07). There was not a significant effect of diet history on tendency of males to be initiators (KW, *p* = .17) or objects (*p* = .71) of intrasexual aggression. No male defended more than one nest tier for the duration of a trial. Post‐trial inspection of birds revealed that one male had sustained a mild beak injury during a trial; this male had paired during the trial. No other injuries or feather loss was noted.

## 4 RESULTS—EXPERIMENT 2

**Figure 5:**
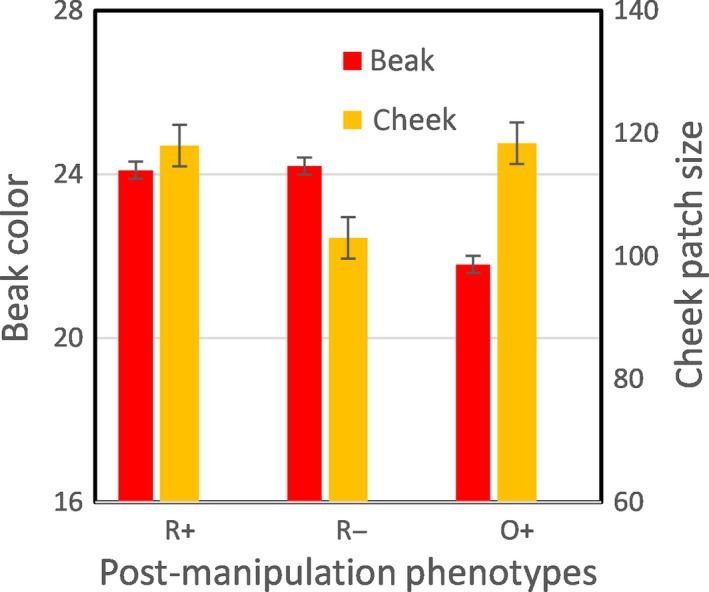
Postmanipulation phenotypes of males in Experiment 2 stimulus sets. Manipulation of traits resulted in significant trait differences within stimulus sets (beak color—LMM: Wald χ^2^ = 214.86, 2 *df*,* p* < .0001; cheek patch size—LMM: Wald χ^2^ = 18.90, 2 *df*,* p* = .0001). R+: males with higher beak color scores and larger cheek patches; R−: males with higher beak color scores and smaller cheek patches; O+: males with lower beak color scores and larger cheek patches. Bars represent marginal means of trait scores (± delta‐method *SE*) for each phenotype across all stimulus sets; random effects (stimulus set identity) made significant contributions to both models

Sixteen test sets resulted in at least one female displaying a preference; in one additional set, no male was preferred. Two females were scored as displaying preferences in five (31%) successful sets, because they reach the criteria for doing so in the same sampling session. For all successful sets, there were no differences in the measured traits of males assigned to treatments prior to phenotype manipulations (Table 5). Postmanipulation beak color score and cheek patch size differed among treatments (Figure 5). Average time to affiliation was 2.5 ± 0.1 days.

**Table 5:** Initial trait scores (prior to manipulation) of males assigned to stimulus sets in Experiment 2

Trait	Assigned treatment	Trait score^a^	Wald χ^2^ (2 *df*)	*p*	95% CI
Age (days)	O+	105.32 ± 6.21	0.75	.69	93.14–117.48
	R−	108.25 ± 6.21			96.08–120.42
	R	104.88 ± 6.21			92.70–117.05
Mass (g)	O+	15.96 ± 0.32	1.46	.48	15.34–16.58
	R−	16.13 ± 0.32			15.51–16.75
	R	16.03 ± 0.32			15.41–16.66
Cheek patch size (mm^2^)	O+	119.01 ± 2.68	0.48	.79	113.75–124.27
	R−	119.99 ± 2.68			114.73–125.25
	R	119.97 ± 2.68			114.71–125.23
Beak score	O+	20.70 ± 0.29	2.73	.26	20.14–21.26
	R−	20.97 ± 0.29			20.41–21.53
	R	20.64 ± 0.29			20.08–21.20

Linear mixed models, with stimulus set number included as random effect. *N*s = 48, except for cheek patch size, where *N* = 42 (due to loss of images).

Marginal mean ± delta‐method standard error, with corresponding CI.

**Figure 6:**
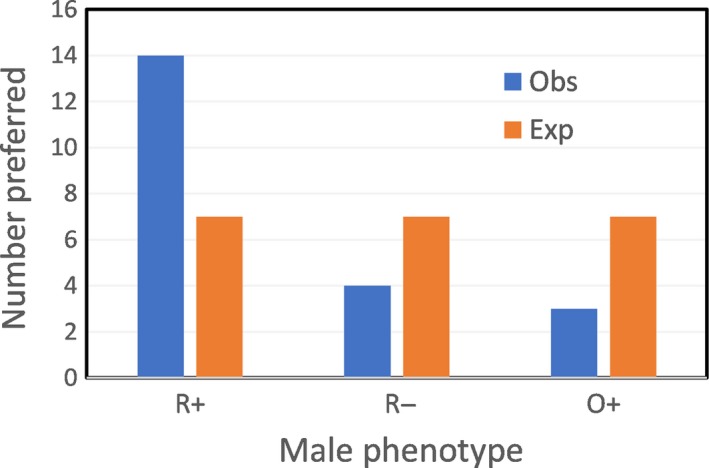
Female preference for manipulated male phenotypes in Experiment 2 (χ^2^ = 18.79, 2 *df*,* p* = .00008; a posteriori comparison of R+ vs. R−: *p* = .0004). R+: males with higher beak color scores and larger cheek patches; R−: males with higher beak color scores and smaller cheek patches; O+: males with lower beak color scores and larger cheek patches

Females displayed significant discrimination among the three male phenotypes; a posteriori comparisons indicate that males with higher beak color scores and larger cheek patches were favored over the other two phenotype combinations (Figure 6).

Very little male–male aggression was observed on the day before females were added to the arena (Day 0: median incidence = 0 bird^−1^ sample^−1^); tendency to initiate aggression did not vary with trial outcome (whether individuals were eventually preferred by females [KW: *p* = .6]). On the day that females were introduced to the arena (Day 1: median incidence = 1.5 bird^−1^ sample^−1^), incidence of aggression increased, but again aggression scores did not vary based on trial outcome (KW: *p* = .5). Across the remaining days, males favored by females were somewhat more aggressive (median = 1.0 incident) than those not favored (median = 0.33 incident) (KW χ^2^ = 3.82, 1 *df*, uncorrected *p* = .051).

Male tendency to initiate aggression was correlated with their tendency to be objects of aggression only on day 0, when there was positive correlation between aggression variables (Spearman ρ = 0.38, *N* = 48, *p* = .007). On day 1 and remaining days, this correlation did not approach significance (*p*s > .5). As in Experiment 1, no male defended more than one tier during a trial. No birds were found to sustain injury during trials.

## 5 DISCUSSION

Our investigation of the influence of several SSTs on relative mating attractiveness in male zebra finches indicates that at least two of them—cheek patch size and beak color—have ornamental value in this population. In Experiment 1, the lack of significant correlations between natural SST expression and tendency to participate in intrasexual aggression (see also Bolund, Schielzeth, & Forstmeier, 2007), combined with the positive relationship between SSTs scores and mate‐getting, indicate that these traits functioned primarily in mate attraction here. Although Experiment 1 produced ambiguous results regarding whether preferences of females for males with large cheek patches and red beaks reflect preference for males reared on the HI diet, the results of Experiment 2 indicate that these preferences are independent of diet.

Several additional lines of evidence indicate that male pairing success in these trials was largely the result of their mating attractiveness rather than intrasexual interference competition: (1) individual males did not defend more than one nest tier (assuring that all males had access to nest sites, which are used in mate attraction in this species) and overall rates and intensity of aggression were low; (2) no evidence of despotic relationships (a negative correlation between tendency to be initiator and object of aggression) among males was observed. As expected, male initiation of intrasexual aggression did correlate with tendency to pair, but (3) much of this aggression reflected male defense of an affiliating female against an unmated “interloper” near his nest site. Finally, (4) results of the second experiment, in which male SSTs were manipulated, provide strong support for the conclusion that female behavior, not male competitive ability linked to SST expression, elicited increased aggression among males.

### 5.1 Diet effects

Rearing diet impacted male mating success in Experiment 1, with HI males about twice as successful in pairing as LO males. Results indicate that treatment differences in mating success were not an effect of differences in size or aggressiveness. The results of SST models predicting pairing success indicate that the larger average cheek patch size of HI males contributed substantially to this treatment effect, but song traits did not systematically predict pairing success (see below). Trait distributions found here correspond with those reported in other studies (beak color—Burley et al., 1992; cheek patch size—Tschirren et al., 2012; song traits—Spencer et al., 2003, 2005).

Previous studies of diet effects on zebra finch song traits, which have typically compared performance of birds reared with ad libitum versus restricted seed access, have been inconsistent in their findings. Our findings are in partial agreement with those of Spencer et al. (2003, 2005), who reported that food restriction during early development resulted in motifs with shorter duration and fewer syllables: here we found a significant diet effect on motif duration, but not syllable number (Table 1). Nevertheless, the inconsistencies among several additional studies regarding both motif duration (no diet effect: Brumm, Zollinger, & Slater, 2009; diet effect: Zann & Cash, 2008) and syllable number (no diet effect: Brumm et al., 2009; Zann & Cash, 2008; diet effect: Spencer et al., 2005) suggest that subtle differences in rearing protocols influence development of song traits (Spencer & MacDougall‐Shackleton, 2011). Some limitations of focusing on isolated song traits for identifying mate preferences for song are discussed below.

### 5.2 Success of SST models

In Experiment 1, we investigated contributions of four SSTs to male pairing success using two statistical approaches. The best overall predictor of pairing success in both approaches was the product of cheek score and beak score (Tables 2 and 4; Figure 4). As ranks of trait scores were positively correlated with their corresponding absolute trait scores, the results of the two procedures are not independent; nevertheless, their close agreement indicates that the result is not a chance statistical artifact, which might result from the exploratory approach used to identify trait synergisms. Thus, this study contributes to a short list of identified cases of multiplicative synergism among male traits influencing mating attractiveness (table 2 in Candolin, 2003). The occurrence of such synergism may indicate that females prefer males whose SSTs are relatively well balanced, such that males with intermediate values on both traits are preferred to those scoring higher on one trait than another other. This could be adaptive when traits communicate different information (implied by the lack of correlation in trait expression) and choice based on both traits has the potential to influence a chooser's fitness. Alternatively, trait interactions might reveal information not provided by individual traits, such as genetic background or individual life history events that dispose individuals to signal above or below average quality on multiple dimensions.

Comparison of the results of modeling based on absolute versus ranked trait scores for all males does not reveal which criterion female zebra finches used here. Nearly 20% of females that displayed heterosexual interest in Experiment 1 failed to reach the pairing criterion, a result that suggests the males available to them may not have passed some threshold of acceptability for some trait or trait combination. However, most females did form partnerships when given a restricted set of options, which is consistent with choice using a relative criterion. This result is not surprising for an opportunistic breeder that experiences high mortality rates in nature (Zann, 1996).

Results of models partitioned by within‐set variation in beak score revealed an intriguing pattern. Under the preference shift hypothesis, we had predicted that females allowed to choose among males in test sets with a very low range of beak scores would emphasize choice for cheek and/or song traits, while those in sets with higher variation would tend to focus more on beak color. Instead, we found that the pattern observed for the full sample (Table 2) was largely the result of the choices made by females exposed to sets containing higher beak score variation (Table 3); by contrast, females exposed to sets with little beak score variation did not display a collective preference for any measured SST. This last finding cannot be explained as an artifact of reduced average variation in expression of other SSTs in these sets, as neither absolute nor ranked beak score correlated with expression of other SSTs. In addition, females exposed to sets with higher beak score variation also appeared to select mates on the basis of a third trait, syllable number (models 9, 13 and 14 in Table 3).

The results obtained by analysis of the partitioned data set have parallels to the findings of Kűnzler and Bakker (2001), who used an elegant virtual mate choice design to study mate preferences of female sticklebacks (*Gasterosteus aculeatus*). In their study, females were initially exposed to videotaped images of all possible 2‐way combinations of males that varied in two traits (throat coloration, courtship display complexity), and relative strength of preference of chooser females was based on the average proportion of time they spent with each videotape. Researchers found no preference for courtship display complexity when both males had dull throats, and a significant preference for throat coloration when both males had either straight or zigzag display; however, preference strength increased when females could choose between a red‐throated male performing a zigzag display versus a dull‐throated male whose display lacked the zigzag. When body size was added as a third variable, the preference strength increased further for those females allowed to choose between males with preferred versus nonpreferred trait values of all three traits. Thus, discrimination between options increased when there was greater difference between options presented, and experimental detection of a preference for one trait (zigzag display) depended on the availability of an option displaying a more strongly preferred trait (red throats).

While it makes considerable sense that mate choice is facilitated by high trait variation, the absence of mate preference for traits that are seemingly easy‐to‐evaluate (cheek patch size, zigzag display) when other traits (beak/throat coloration) show little variation begs for explanation. One possibility is trait amplification, or the tendency of one trait to improve perception of another (Candolin, 2003). Sticklebacks may naturally illustrate this phenomenon, in that the zigzag display is thought to improve female perception of ventral throat coloration by exposing it more clearly to their view (McLennan & McPhail, 1990). In Kűnzler and Bakker's study, however, the size of the throat patch was enlarged to be clearly evident even in the absence of the zigzag display. Trait amplification seems unlikely to apply to our results as well, because beak and cheek are discrete traits and are visible simultaneously. We suggest an alternative possibility, namely that certain male traits serve as “linchpins” that elicit expression of consensus mate preferences of females. According to this hypothesis, when males in the choice pool lack sufficient variation in a key trait, the typical variation displayed by other SSTs may not provide sufficient information about male quality for females to agree on which males are the most promising mates.

The linchpin SST concept is consistent with the trait synergism hypothesis, in that males that score relatively high on the product of two or more SSTs—one of which serves as a linchpin—may exceed some threshold value that makes them attractive to a large number of females. When such outstanding males are not available, females may be more responsive to their own individual circumstances and needs, leading them to choose mates based on somewhat idiosyncratic criteria, which would reduce the intensity of sexual selection. Females may, for example, increase choice based on genetic complementarity (Mays et al., 2008) or behavioral compatibility (Fox & Millam, 2014; Ihle, Kempenaers, & Forstmeier, 2015).

Evidence that choosers base mating decisions on the interaction between two phenotypically uncorrelated traits has important practical and conceptual implications. One consequence of this phenomenon is that it increases the likelihood that studies that exclude some traits used in mate choice will fail to uncover evidence for traits that are included and for which choosers do have preferences (type II statistical error; Galván, 2010; Hamilton & Sullivan, 2005). To avoid this outcome, it is desirable to include as many traits as possible in empirical studies focusing on sexually selected traits, and to seek evidence for trait interaction effects. The occurrence of mate choice for trait synergisms may also be pertinent to interpretation of studies that explore population differences in SST expression and preferences for them. For example, the reported tendency of choosers to focus exclusively on the single trait that is locally most variable (Dale & Slagsvold, 1996; Reid & Weatherhead, 1990) may underestimate the occurrence of choice for multiple traits if possible trait interaction effects are not considered. Finally, the occurrence of trait interactions influencing mate preferences complicates interpretation of findings regarding repeatability and heritability of mate preferences for single traits (Bell, Hankison, & Laskowski, 2009). Trait interactions and the possibility that some SSTs serve as linchpins in eliciting consensus preferences have implications for measurement of the heritability of mate preferences, and for understanding the strength of selection on and persistence of heritability of SSTs (Prokuda & Roff, 2014) and the lek paradox (Kotiaho, LeBas, Puurtinen, & Tomkins, 2008).

Although studies on other species have reported synergisms involving traits communicated by more than one sensory modality (table 2 in Candolin, 2003), in this study it appears that song traits did not interact synergistically with cheek and beak scores (i.e., models 13 and 14 in Table 3 are weaker than models 11 and 12). However, bird song is a very complex trait or set of traits (Catchpole & Slater, 2008; Gil & Gahr, 2002) and measures of isolated components may not well approximate how songs are evaluated by choosers. For any given species, it is important to explore how song components may be evaluated by females, including the possibility of trait synergisms and trait composites (Badyaev, Hill, Dunn, & Glen, 2001) before concluding that females lack consistent preferences for song traits or that trait synergisms involving acoustic and visual information are absent. For zebra finches, several candidate song trait components have been identified in addition to those studied here (Riebel, 2009), but their possible interactive effects have not been investigated. The search for reliable predictors of possibly interacting song traits may be advanced by partitioning traits into those that largely reflect male motivation/physiological state (and thus have lower repeatabilities) and traits that may serve as long‐term quality indicators (as indicated by higher repeatabilities).

## CONFLICT OF INTEREST

None declared.

## AUTHOR CONTRIBUTIONS

NTB conceived project, contributed to data collection, analyzed results, drafted manuscript; EH contributed to data collection (primary collector of behavior data, Experiment 1) and comments on manuscript; CS contributed to project conception, data collection in both experiments, and manuscript preparation.
